# Forms and Functions of the Electric Discharge

**Published:** 1882-03

**Authors:** 


### FORMS AND FUNCTIONS OF THE ELECTRIC DISCHARGE.

On the Monday evening during the meeting of the British Association, Mr. Spottiswoode, Pres. R. S., delivered a lecture on "The Electric Discharge: its Forms and Functions," in the course of which he said: We may now fairly ask whether the phenomena which we have been studying have any counterpart in the larger operations of nature which are going on around us, and whether the conclusions to which we have been led afford any explanation of observed facts.

Many natural phenomena doubtless fundamentally depend upon electricity; how many we hardly yet know. But there are two in particular, namely, lightning and the aurora—which are unquestionably electrical, and whose correspondence, with the spark proper, and with the discharge in rarified gases respectively, has often been noticed. In the thundercloud we have an aggregation of aqueous, particles small enough to remain, temporarily at least, suspended in the air. All of these, it would appear, are similarly electrified, and by their mutual repulsion, are restrained from further coalescence. By their presence the ground below the cloud becomes inductively electrified in the opposite sense, and as soon as the cloud by its motion comes within sparking distance, or by an increase of its charge attains sufficient tension, a spark discharge takes place, which as we have seen above is a flash of lightning. A similar action may naturally take place between two clouds oppositely electrified.

The electrical tension required for a flash of lightning is, of course enormous. It has been calculated that, in order to produce directly from a battery of the most favorable construction a spark of 42 inches, equal to that given from my great induction coil, from 60,000 to 100,000 cells would be necessary, while for a flash of lightning a mile long not less than 3,500,000 cells would be required. There is, moreover, another kind of lightning to which the discharge in our vacuum tubes offers, to say no more of it, considerable analogy—namely, that commonly known as ball lightning. The appearance of ball lightning is described as that of a luminosity or ball of fire moving generally toward the earth, in a direction more or less oblique, and disappearing in most cases before reaching the ground.

In some tubes, the exhaustion of which is very moderate, say having a pressure of several millemeters of mercury, it happens not only that the blocks of light termed entities by Mr. De La Rue, are formed, but also that these entities travel along the tube from the immediate neighborhood of the positive terminal to a finite distance in the direction of the negative, and then disappear. It would seem not unreasonable to suppose that ball lightning is due to conditions not dissimilar to those of such tubes—namely, to a discharge occurring in the upper regions of the air, at an elavation of, perhaps, 20 miles, more or less, where the pressure is very moderate—that is to say greater than that under which an auroral-like display could take place, and yet less than that which would give rise to a true spark or ordinary flash of lightning.

We have not unfrequently present in regions at moderate elevations, say from 20 to fifty miles, all conditions necessary for the production of an auroral display. And not only so, but our experiments enable us to determine, at all events approximately, some limits of elevation within which this phenomenon can occur, and thereby to check the very divergent estimates of those who have observed it.

Estimates of the altitude at which the auroral discharge takes place have been made from simultaneous observations at different points, and these have ranged up to 50 or 60, and even to 281 miles But even the lowest of these appears to be inprobable. The pressure at which the resistance of air is least, is a little less than 0.4 of a millimeter of mercury ; and the corresponding elevation is about 38 miles. A vacuum tube measured by hundred thousandths of an atmosphere would correspond to an elevation of a little more than 81 miles. Though a hydrogen vacuum at this pressure Mr. De La Rue failed to obtain a discharge with 11,000 cells ; and he adds that,

"it may be assumed that at this height the discharge would be considerably less brilliant than at 38 miles, should such occur."

It seems to me a well ascertained fact that in high latitudes there are fewer thunderstorms and more auroras than in lower latitudes. This fact points to the conclusion that, after a disturbance, the redistribution of atmospheric electricity is effected by one process or by the other, according to, or rather in consequence of the meteorological differences between arctic, temperate, and tropical regions.—*Scientific American.*

